# Impact of USMLE Step-1 accommodation denial on US medical schools: A national survey

**DOI:** 10.1371/journal.pone.0266685

**Published:** 2022-04-14

**Authors:** Kristina H. Petersen, Neera R. Jain, Ben Case, Sharad Jain, Lisa M. Meeks

**Affiliations:** 1 Department of Biochemistry & Molecular Biology at New York Medical College, Valhalla, New York, United States of America; 2 Centre for Health Education Scholarship, The University of British Columbia Faculty of Medicine, Vancouver, British Columbia, Canada; 3 Department of Family Medicine, University of Michigan Medical School, Ann Arbor, Michigan, United States of America; 4 Department of Medicine, University of California, Davis, School of Medicine, Sacramento, California, United States of America; 5 Center for a Diverse Healthcare Workforce, University of California Davis, Sacramento, California, United States of America; Ohio State University, UNITED STATES

## Abstract

**Introduction:**

In 2019, 4.6% of US-MD students self-identified as students with disabilities (SWD); many of these students will require accommodations on the USMLE Step-1 examination. Given the high-stakes nature of Step-1 for medical school advancement and residency match, SWD denied accommodations on Step-1 face considerable consequences. To date no study has investigated the rate of accommodation denial and its impact on medical school operations.

**Methods:**

To investigate the rate of accommodation denial and evaluate whether Step-1 accommodation denial impacts medical school operations, a 10-question survey was sent to Student Affairs Deans and disability resource professionals at all fully-accredited US-MD granting programs. Two open-ended questions were analyzed using qualitative content analysis.

**Results:**

Seventy-three of the 141 schools responded (52%). In the 2018–2019 academic year, 276 students from 73 schools applied for Step-1 accommodations. Of these, 144 (52%) were denied. Of those denied, 74/144 (51%) were delayed entry into the next phase of curriculum and 110/144 (76%) took the Step-1 exam unaccommodated. Of the 110 who took Step-1 without accommodations, 35/110 (32%) failed the exam, and 4/110 (3%) withdrew or were dismissed following exam failure. Schools reported varied investments of time and financial support for students denied accommodations, with most schools investing less than 20 hours (67%) and less than $1,000.00 (69%). Open-responses revealed details regarding the impact of denial on schools and students including frustration with process; financial and human resources allocation; delay in student progression; lack of resourcing and expertise; and emotional and financial burdens on students.

**Discussion:**

Step-1 accommodation denial has non-trivial financial, operational, and career impacts on medical schools and students alike. The cause of accommodation denial in this population requires further exploration.

## Introduction

In 2019, 4.6% of allopathic medical students disclosed disabilities, reflecting a 69% relative increase in disclosure of disability since 2016 [1, 2]. Responding medical schools reported that students with disabilities [SWD) utilized additional time accommodations on in-house standardized exams [2]. Students who received additional time on standardized exams administered in a medical school curriculum will likely require similar accommodations on the United States Medical Licensing Examinations [USMLE), including Step-1. However, concerns exist about student access to accommodations on USMLE exams [3–8]. Despite the importance of these concerns, little scholarly attention has been paid to the matter, including gaining an understanding of the effect of denials on school operations and on medical student progression. Collecting information about the effects of accommodation denials on schools and students would facilitate better understanding of, and illuminate potential barriers to, accommodation access.

Researchers, students and medical associations affirm the value of students with disabilities [SWD) as an important part of a diverse physician workforce that represents the patient population [3, 9–11], while accrediting bodies and associations offer guidance or mandates guiding the inclusion of this group of students [3, 12, 13]. Despite these stated commitments, studies suggest that SWD may still face barriers in the medical education learning environment. For example, students report that the process of applying for USMLE accommodations is arduous, requiring many hours to complete the application and gather required documentation, which often goes beyond the threshold of documentation required for medical school accommodations [4]. Indeed, obtaining approval for accommodations on USMLE exams can be difficult, as evidenced by recent litigation [14–17]. SWD score lower on Step-1 than their non-disabled peers [6, 18, 19], A recent multi-site study suggested that approximately 25% of students with disabilities in their sample were approved for accommodation on Step-1. Students in this study who received accommodation on Step-1 performed better than those without accommodation, by an average of 6 points. The authors postulate that for some, failure to receive accommodations on Step-1 may necessitate a leave of absence to appeal the decision and/or provide time for additional test preparation [6]. Given this, the inability to obtain accommodations on Step-1 likely presents a barrier to medical student progression, disrupting a student’s educational pathway, or requiring a leave of absence (LOA). Moreover, students aware of the difficulties associated with the Step-1 accommodation application process may choose to take the exam unaccommodated, despite knowing that the score will not represent their full abilities.

### Step-1 failures and consequences

Data obtained from the National Board of Medical Examiners’ (NBME) annual reports of allopathic medical students in the US and Canada show that in the 2018 and 2019 calendar years, 5% and 4% of students, respectively, failed the Step-1 examination [20]. Step-1 failure or obtaining a score that does not accurately represent the student’s knowledge due to lack of accommodations comes at a cost. Passing Step-1 is a requirement to enter or continue the clinical portion of the curriculum and to graduate from MD-granting schools [21–24]. Furthermore, students who have delayed entry to the clinical phase of their education must explain this on their residency application. Therefore, Step-1 failure in conjunction with the subsequent delay to entering clinic can negatively impact a student’s prospects for the residency match [25, 26]. Although the NBME allows students to take Step-1 six times, most medical schools limit students to three attempts [27], after which students are forced to withdraw or are dismissed, resulting in “debt without degree,” a high-risk recipe for diminished well-being in a population that is already at increased risk of distress [28].

Moving Step-1 administration to after the clinical year may benefit some students; preliminary data suggest fewer students fail and mean scores are higher [29–31]. The conversion of the USMLE Step 1 from a 3-digit score to pass/fail, planned for January 2022 may also benefit some students by reducing the anxiety that accompanies test-taking [32, 33]. However, these changes do not fully address disability-specific barriers and could potentially create new barriers for SWD. For example, SWD who do not receive accommodations and ultimately fail Step-1 may experience an increase in emotional distress and financial debt with limited time to retake the exam [33, 34]. With less time to engage in the application process during clinical years, SWD may also be less motivated to apply for accommodations altogether, understanding the time commitment and low rate of success on requests. Making the exam pass/fail does not address lack of access to the exam, or the impact on SWD who may fail the exam due to time-related barriers.

While the impact of accommodation denial on students is often discussed, no study to date has investigated the impact of Step-1 accommodation denials on medical school operations. This study aims to understand: 1) the school-based financial and resource implications following Step-1 accommodation denial, and 2) the proportion of students who request and receive Step-1 accommodations and their subsequent progression through the MD program. We also collected qualitative data on medical school administrators’ experiences with the Step-1 accommodation process. This information is critical to understanding the collective impact of accommodation denials on medical school operations.

## Methods

Between June and October 2020, a survey was sent to Student Affairs (SA) Deans at fully accredited Liaison Committee for Medical Education (LCME) allopathic medical schools. Disability resource professionals at all schools were provided a copy of the survey to assist SA Deans in gathering information. Like previous studies [1, 2, 35, 36], we excluded schools with a *provisional* or *preliminary accreditation*, those on *probation*, or those with *exempt* status (n = 15). The resulting school sample size was 141. Responses were collected from June to October 2020, with an email reminder sent in July, August, and September. This study was deemed exempt by the University of Michigan Institutional Review Board as data were fully anonymized and only shared in aggregate.

### Survey instrument

A 10-question survey was developed by the authors (KHP and LMM), seeking data about the impact of Step-1 accommodation denial on medical school operations, including administrative and financial resources allocated to support SWD who are denied accommodation. In measuring financial and administrative resources, SA Deans were asked to select from a range of times [0–10 hours; 11–20 hours; 21–30 hours; 31–40 hours; and greater than 40 hours] and costs [between $0-$1,000; $1,001-$5,000; $5,001-$10,000, and greater than $10,000]. Two free-response questions asked SA Deans to comment on institutional and student impact following Step-1 accommodation denials. Although administrators cannot speak on behalf of SWD, we included this question to seek SA Deans’ observations of student impact and to inform future avenues for research. We also gathered data on the number of students who requested and received accommodations on Step-1 in the 2018–2019 year including questions about the number of SWD who failed Step-1, took a leave of absence (LOA) or who were dismissed due to Step-1 failure. The 2018–19 academic year was selected to avoid anomalies caused by COVID-19. [S1 File].

The survey content was pilot tested by three medical school SA deans who were not institutional respondents for the final survey. The survey was refined for content and clarity following their feedback.

### Data analysis

Responses were linked to the 2018 AAMC Organizational Characteristics Database. Data included: medical schools’ region, ownership, financial characteristics, and class size. All organizational data, except class size, were categorical. One investigator (BC) developed categories for class size using national medical school cohort means and ranges as a guideline. Class size categories were defined as small [<100 students), average [100–200 students), and large [>200 students). To assess the representation of survey data, respondents were compared with non-respondents using Pearson’s chi-square and Fisher’s exact tests. Data analysis was conducted using IBM SPSS Statistics Version 26.

Responses to the two open-ended questions were analyzed qualitatively (NJ). Participant responses varied in length, from several words to multiple paragraphs. An inductive content analysis process of open coding, grouping, and categorizing was followed to identify key messages from this qualitative data and cluster them into categories [37]. The research team reviewed the groupings to reach agreement on final categories.

## Results

Seventy-three of the 141 schools completed the survey (52% response rate). No associations were found between institutional characteristics, disability disclosure structure, and class size across all outcome measures.

### Accommodation requests, denials, and progression

**Fig 1:**
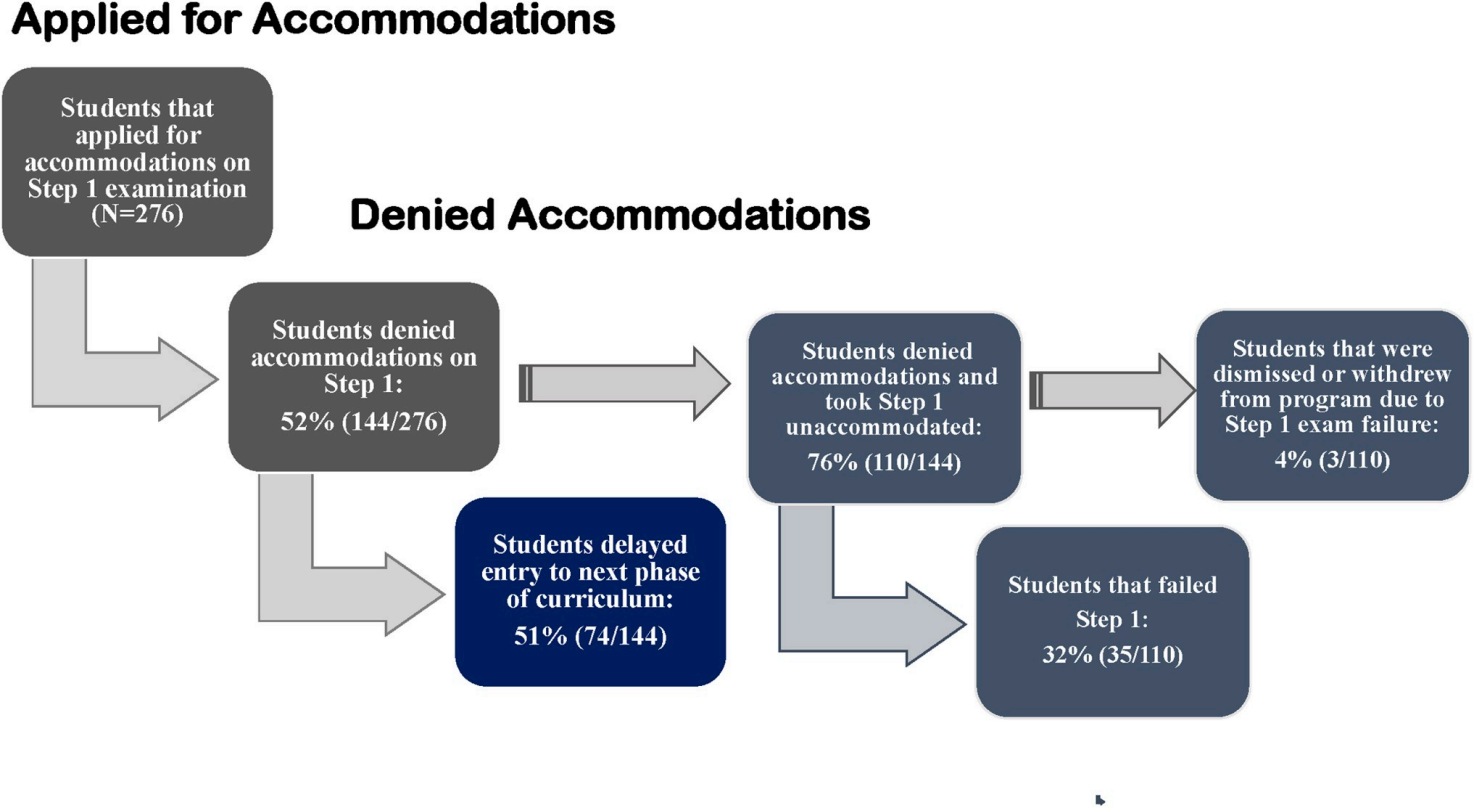
Progression of students with disabilities denied accommodations on Step 1 examination.

For the academic year 2018–2019, the 73 schools that responded to the survey collectively reported that 276 students applied for accommodations on the Step-1 exam. Of these, 144 [52%), were denied accommodations. Of the 144 students denied, 74 (51%) were delayed entry into the next phase of their program because of the denial. In sum, 110 (76%) of the 144 denied students took the Step-1 examination without accommodations; of these, 35 (32%) received a failing score and 4 (3%) withdrew or were dismissed from their program due to the failing score (Fig 1).

### Resource allocation for students denied accommodation

**Fig 2:**
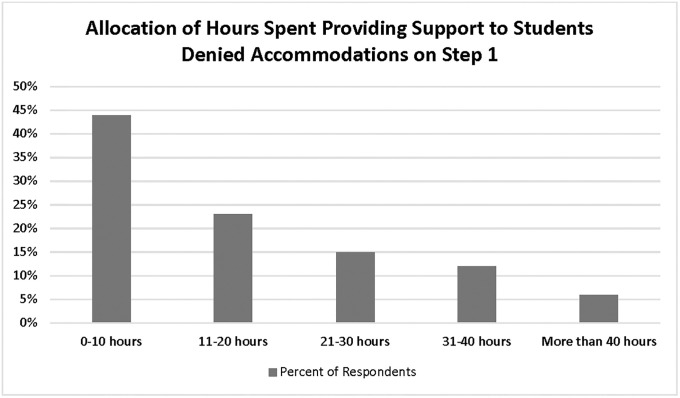
Allocation of hours spent providing support to students denied accommodation on Step 1 examination [N = 73).

Schools were asked to estimate the total number of hours and financial resources committed to supporting students who were denied accommodations on Step-1 (Fig 2), including deferring and rescheduling clerkships, monitoring practice exam scores, organizing appointments and study strategies, writing Step-1 extension letters, supporting accommodation appeals, and promotions committee advocacy. Most schools reported providing between 0–10 hours (44%) and 11–20 hours (23%) of administrative and academic support.

Financial implications for academic support resources provided by institutions varied (Table 1). When asked about the financial resources allocated to academically support students who were denied accommodations on Step-1, the majority (69%) reported allocating between $0-$1,000, followed by 11% reporting $1,001-$5,000, 11% reporting $5,001-$10,000, and 9% reporting a financial expenditure of greater than $10,000. Schools were also asked to estimate the total dollar amount of any financial resources spent by the institution to support living expenses and continued coverage of insurance, etc. for students who were denied accommodations on Step-1 and unable to continue in the curriculum. The majority (74%) reported investing $0-$1,000, while 10% of schools reported spending greater than $10,000.

**Table 1:** Financial resource allocation for students denied accommodations on Step 1.

Institutional Resources	$0-$1,000	$1,001-$5,000	$5,001-$10,000	$10,001-$15,000	More than $15,001
**Financial Support Academic Needs [US dollars) N = 73 respondents**	69% [50)	11% [8)	11% [8)	5% [4)	4% [3)
**Financial Support for Student Living Expenses and Insurance [US dollars) N = 73 respondents**	74% [54)	11% [8)	5% [4)	3% [2)	7% [5)

### Institutional impact

36 schools provided open-text responses regarding the institutional impact of Step-1 accommodation denials. Three categories were identified: *financial and human resource impact*, *staff frustration*, *and institutional implications of student progress delays*. Responses suggested that perceptions of institutional impact are moderated by the *level of expertise*, *resourcing*, *and preemptive attention* to the Step-1 accommodations process.

The most common response (11/36) addressed the ***financial and human resource impact*** of denied accommodations in the form of staff time to assist in assembling appeals, develop new exam-taking strategies without accommodations, and provide students with emotional support. One respondent explained:

*The process of supporting students through the accommodation’s application process has been made very time consuming by denials of accommodations by USMLE*. *The person hours required per applicant is significant and places strain on staff with very full caseloads*.

(R80)

Two schools noted the strain of limited human resources represented a significant investment in a few students. Two schools reported that denials led to increased tutoring costs and the need to hire additional student support staff.

Over 25% (10/36) of respondents indicated ***staff frustration*** with the perceived high denial rate and time-consuming process of requesting accommodations. Frustrations were fueled by perceptions of an unjust and invasive process, observing students with long histories of disability denied accommodations, and the time-consuming process to apply and receive a decision.

One respondent stated:

*Our students are disheartened and traumatized by the entire experience*. *It is beyond frustrating to witness the injustice*.

(R27)

The ***impact of student progress delays*** was also noted (10/36). Schools reported extending student Step-1 pass deadlines, increasing study timelines, and offering LOA’s to prepare for accommodation appeals (e.g., obtaining additional disability documentation) and for prolonged time-to-decision. This impacted the schools’ ability to plan for class numbers, resource allocation and clerkship capacity. Unfortunately, two schools explained this also impacted their graduation rates.

Several schools described less institutional impact than others. 11 responses indicated this may reflect the ***relative impact of resourcing and the disability-related expertise for assisting students with their application***, suggesting that the level of preemptive support available for SWD may moderate how respondents characterized the institutional and student impacts of Step-1 denials. For example, one school with a dedicated disability resource professional for health sciences did not characterize 10–15 hours spent per student application as burdensome. Six schools described preemptive efforts to minimize institutional and student impact, including developing stronger support processes within the school, providing application timelines, counseling students on whether to apply, and supporting students for the possibility of denial. In the absence of resources and expertise, outcomes were predictably different. Two schools that utilized central university disability resources noted that the lack of available expertise in the Step-1 application process deterred students from applying for accommodations and reduced the effectiveness of submitted applications. However, two schools stated difficulties related to accommodation denials had attenuated over the last two years. One school indicated there was no institutional impact because students that were denied Step-1 accommodations ultimately passed and graduated.

### Student impact

Of the 73 survey respondents, 43 responded to the open-ended question regarding their observations of the impact of the Step-1 accommodation process on their students. Responses comprised 5 categories: *emotional toll*, *lost time*, *impacted performance*, *financial burden*, and *choosing not to apply*. Although the question focused on the impact of denials, some respondents spoke to the wider negative impact of the Step-1 accommodation request process on students, which was then compounded by a denial.

Over half (24/43) of respondents discussed the ***emotional toll*** on students. Terms such as fear, anger, devastation, anguish, demoralizing, traumatizing, suffering, frustration, stressful, and distressful were used to characterize this impact. Respondents attributed these terms to the challenges of putting together an application, which required a high degree of vulnerability, and the impact of forging ahead without accommodations or into an appeal. One respondent encapsulated this experience:

*It has been very stressful and scary for students*, *making a high stress and high-pressure time even more daunting*.

(R16)

Seven suggested the application process affected students’ mental health, triggering anxiety and depression. Importantly, three responses noted that denials shook students’ confidence and caused them to question their disability status. This emotional toll was closely connected to the other themes that follow.

Just under one third of respondents (14/43) reported ***lost time***, attributed to the “*labor-intensive*” (R13) process of applications and appeals including time to obtain suitable documentation, long decision times, and delayed exams. This experience was closely connected to the emotional toll and time delays that removed students from their cohort and slowed their progress to graduation. Two schools explained that students strategically prepare as if their request will be denied and try to adapt without accommodations. This required additional time and elevated access to support services including exam preparation support.

***Impacted performance*** was similarly frequent (14/43). Nine respondents believed denials led to underperformance on the exam and other coursework due to lack of equal access to Step-1, increased stress, and lost confidence. As one respondent explained:

*They are defeated before they even take the exams*, *as they know that accommodations lessen the barriers that their disabilities present*.

(R44)

Five respondents described possible exam failure, dismissal, or withdrawal because of accommodation denial. Five respondents also suggested that underperformance resulted in decreased competitiveness for residency. Even if students passed, they may not match into their preferred specialty due to underperformance.

Seven respondents spoke to the ***financial burden*** on students, which one described as *“often significant and disproportionate*” (R64). This burden comprised costs to obtain additional disability documentation, paying for remedial preparation programs, and the increased debt burden from extended living costs due to LOA and delayed graduation.

Almost 20% of respondents (8/43) described students ***choosing not to apply*** for accommodations altogether or forgoing appeal processes due to the associated costs and perceived low success rate informed by historical accounts. One respondent explained:

*A majority of students receiving university-approved accommodations are interested in pursuing an NBME accommodation request*, *but many choose not to pursue a request due to*: *1) the potential cost to secure an updated psychoeducational or neuropsychological evaluation*, *if needed 2) the known history that very few accommodation requests are approved*.

(R10)

Thus, respondents explained, the perception of likely denial deterred many students from pursuing accommodations in the first place and others from pursuing appeals.

## Discussion

Like other studies [6, 18, 19], our results show that the majority of SWD eventually graduate from medical school but with significant impact on student progression. To our knowledge, ours is the first study to quantify the number of students denied Step-1 accommodations and delineate the pathways that follow. In this study over half of SWD who applied for Step-1 accommodations were denied, and over half of those denied accommodations delayed entry into the next phase of the curriculum. Ultimately, nearly one-third (32%) of SWD who were denied and took the exam without accommodations failed Step-1. This is particularly notable when compared to the overall Step-1 failure rate of 4–5% during the 2018–2019 academic year [20].

In addition, over 25% of qualitative respondents described the impact of student progress delays that caused administrative and logistical disruption, including around clerkship enrollment and capacity. Delaying progression takes students away from the support of their cohort, requires alterations in scheduling by the institution, requires additional explanation on residency applications, necessitates student financial investment, and may postpone graduation and entry into residency. This may result in a significant impact on students’ mental health [removal from their support system), make them less competitive for the match [given the delay to graduation and the need to explain their disability-related delay), and place students in extraordinary debt, above and beyond the amount budgeted for medical school.

Our results also suggest that medical schools are impacted financially and experience administrative time burdens when students are denied accommodations on the Step-1 examination. Almost 70% of institutions spent $0-$1000 to academically support students who are denied Step-1 accommodations, while 74% spent $0-$1000 to provide support for living and/or insurance expenses during “holding periods” as students engaged in appeals or waited to retake the exam. While these low estimates were initially surprising, qualitative responses revealed that some schools encountered increased tutoring and staffing costs, which may not have been captured in the numerical values provided in response to survey questions. Furthermore, reported costs may underestimate actual expenses, as respondents may have omitted budgeted services already embedded in the support system. Conversely, some schools may not allocate additional resources to support SWD in this situation. Students may also have to cover expenses not reflected in our study [e.g., rent, tutors, board preparation). Nonetheless, these data indicate uneven allocation of resources across institutions nationally and suggest frequent under-investment in this area [3]. These findings demonstrate the need for parity in medical school support of SWD who require Step-1 accommodation. Standardization of investment and allocation of resources to support SWDs applying for USMLE accommodations across institutions is necessary to ensure all students have equitable access to expert disability support [8, 38, 39]. Our results also highlight the need for institutions to invest in disability resources more generally, to relieve part of the burden of application from the student. Financial support to update documentation, release for time to be reevaluated and to prepare the application are also needed. Ideally, the process for applying for NBME accommodations would parallel that of the medical school, making the transition from school based to board exam-based accommodations easier on all parties.

Many institutions reported a significant investment of time addressing denials. Over half of respondents spent more than 10 hours, while 18% reported over thirty hours of direct support. Qualitative responses revealed staff frustration with a burdensome accommodation request process requiring a significant time investment for staff. Although the survey focused on student support following Step-1 accommodation denials, qualitative responses provided broader information. The veracity of these staff members’ qualitative responses is reflected in publications that address how to effectively support students seeking USMLE accommodations preemptively and post-denial [4, 7, 40]. Preemptive support is not fully captured in this study and could be considerable across departments [e.g., supporting application preparation, developing a detailed institutional letter of support, developing test-taking strategies prior to denial), and likely would vary greatly between institutions [4, 38, 39].

Although our survey did not query appeals, qualitative responses illuminated barriers including the application process, time, and resource availability. Barriers to appeal included lack of expert disability resource professional staff to help students frame and support requests for appeal, cost to update disability documentation, likelihood of further delays to the clinical portion of the curriculum, and institutional advice to forgo an appeal based on perceived lack of application success. Addressing these barriers could begin to address student hesitation about applying for Step-1 accommodations.

Our findings add depth to existing reports of an arduous Step-1 accommodation request process coupled with SWD’s limited time during medical school [4, 41]. Consequences are heightened by reports of an emotional, temporal, and financial toll for students engaged in accommodation request and denial processes. While not the focus of this study, the significant impact of Step-1 accommodation denial on students, as reported by SA Deans, requires further exploration.

As demonstrated in this study’s findings, failure to receive accommodation on Step-1 places students on a non-optimal pathway. However, schools can and must support students to improve the quality of their accommodation requests. Extensive guidance on how to improve support has been outlined elsewhere [4, 7]. While these outlined steps improved school-based services can support students to submit better quality and more timely requests for accommodation, this process remains labor and time intensive [4, 40]. As noted in qualitative responses and as described in other studies, SWD in medicine already have limited time [41]. These same sentiments were expressed in a recent American Medical Association report that suggests, among other things, “These processes [NBME Step 1 Accommodations Requests] should require neither proof of accommodation nor proof of poor academic performance prior to the time at which a need for accommodation was requested.” [8] Given our findings, coupled with historical knowledge of barriers to access, an examination of the USMLE accommodation request process is in order to identify mechanisms that streamline student requests.

This study has limitations. First, our survey only analyzed requests and denials over one academic year and therefore does not capture trends. Second, the survey did not address the resources students and institutions expended toward an initial application for accommodation, only those after denial. Some schools report spending considerable resources prior to denial. Third, this study focused only on students who applied for Step-1 accommodations; our findings could underestimate the impact by omitting students who chose not to apply due to a perceived burdensome process with a likelihood of poor outcomes. We also did not assess student performance outside of Step-1, limiting our understanding of performance issues contributing to Step-1 failure. Perceptions of student impact were gathered from SA Deans capturing their collective insights into student experiences. To understand student impact more fully, direct research with students is necessary. Finally, because this survey was voluntary and captures only 52% of LCME fully accredited medical schools, there was a potential response bias toward schools more impacted by Step-1 denials or those with more SWD, as they may have greater concerns about the provision of accommodations.

### Summary

To our knowledge this is the first study to investigate the impact of Step-1 accommodation denials on medical school operations. Findings indicate that Step-1 accommodation denials have non-trivial consequences for medical schools and SWD alike. These financial and administrative burdens placed on medical schools may unintentionally work against commitments to inclusion by disincentivizing the admission of SWD for fear of downstream consequences associated with Step-1 accommodation denial (e.g., taking a leave of absence, delayed graduation, and failure of Step-1).

Future research should explore barriers to the Step-1 accommodation application process, the disconnect between institutionally approved accommodations and those afforded on Step-1, students’ rationales for deciding whether to appeal, and the impact of the NBME process and accommodation denials on student’s medical school experience and wellbeing. Direct research with students to understand their lived experience of the NBME accommodation application experience, including its impact on their medical school experience, is necessary.

## Supporting information

S1 Data (XLSX)

S1 File (PDF)
