# Supplementary material for: Impact of USMLE Step-1 accommodation denial on US medical schools: A national survey
Source: PLoS One. 2022 Apr 14;17(4):e0266685. doi: 10.1371/journal.pone.0266685 (PMC9009603; doi:10.1371/journal.pone.0266685)
Supplement: S1 File — (PDF) [file pone.0266685.s002.pdf]

For the purposes of the study, the 2018-2019 academic year is considered to be *July 1, 2018-June 30, 2019.*

Default Question Block

1) Which of the following best represents the structure for student disability determination at your institution?

- ○ The School of Medicine employs a disability resource professional who reviews requests for accommodation
- ○ The School of Medicine utilizes a disability resource professional who works for the health science campus broadly
- ○ The School of Medicine utilizes an internal committee of faculty and administrators to make determinations about disability status and accommodations
- ○ The School of Medicine's dean of students makes determinations about disability status and accommodations
- ○ The School of Medicine utilizes the assistance of our undergraduate disability services office, ***with a specific liaison for medicine***

- ○ The School of Medicine utilizes the assistance of our undergraduate disability services office ***without a liaison***
- ○ [ ] Other

2) In the 2018-2019 academic year, how many students with disabilities applied for exam accommodations for the USMLE Step 1?

If unknown, please provide your best guess

Enter number [ ]

3a) How many that applied for examination accommodations on USMLE Step 1 were denied by the NBME?

Enter number [ ]

3b) For those denied accommodations on Step 1, how many students delayed entry into the next phase of your program?

For example: To attempt appeals, increase study time, or take a leave of absence in general?

Enter number

3c) Of the students who applied for but were denied accommodations on Step 1:

How many took the exam unaccommodated?

Of those who took Step 1 unaccommodated, how many failed the exam?

Of those who failed Step 1, how many were dismissed or withdrew solely due their Step 1 failure? (do not include students who struggled with academic or professionalism issues that impacted their dismissal).

4) Please estimate the **total number of hours student support offices (Student Affairs Deans and Staff, Diversity, Learning Specialists, etc.)** spent supporting students who were denied accommodations by the NBME.

Hours may include: deferring and rescheduling clerkships, monitoring practice exam scores, in-person appointments, study strategies, writing Step 1 window extension letters, supporting students at promotions committees, etc.

- ○ 0-10 hours
- ○ 11-20 hours
- ○ 21-30 hours
- ○ 31-40 hours
- ○ [ ] More than 40 hours (enter estimated number of hours)

5) **Please estimate the total dollar amount of any institutional financial resources spent to academically support students who were denied accommodations by the NBME.**

Expenses may include study resources, tuition for study programs, internal or external tutoring services, independent study, research, etc.

- ○ 0-$1,000
- ○ $1,001-$5,000
- ○ $5,0001-$10,000
- ○ $10,001-$15,000
- ○ [ ] More than $15,001 (enter estimated dollar amount)

6) **Please estimate the total dollar amount of any financial resources spent to support living expenses and continued coverage of insurance, etc.** for students who were denied accommodations by the NBME and who were unable to continue in the curriculum.

This includes students who took a leave of absence. Expenses may include housing, off-setting financial aid income, cost of continued insurance, etc.

- ○ 0-$1,000
- ○ $1,001-$5,000
- ○ $5,001-$10,000
- ○ $10,001-$15,000
- ○ [ ] More than $15,001 (enter estimated dollar amount)

7) Does your institution offer a terminal Master's degree for students who do not wish to, or are ineligible to continue studies beyond the pre-clinical years?

- Yes
- No

8a) Do you believe that a terminal master's degree would be beneficial for students who choose to withdraw or are ineligible to continue beyond the pre-clinical years because of a Step 1 failure?

- Yes
- No

8b) Is your program planning to add a terminal Master's Degree?

- Yes
- No

9) Please share any additional information about **how your INSTITUTION has been impacted** by the denial of accommodations on USMLE Step exams.

10) Please share any additional information about **how your STUDENTS have been impacted** by the denial of accommodations on the USMLE Step exams.

Powered by Qualtrics
